# Efficacy of radiotherapy for gastric bleeding associated with advanced gastric cancer

**DOI:** 10.1186/s13014-021-01884-5

**Published:** 2021-08-23

**Authors:** Joongyo Lee, Hwa Kyung Byun, Woong Sub Koom, Yong Chan Lee, Jinsil Seong

**Affiliations:** 1 Department of Radiation Oncology, Yonsei Cancer Center, Yonsei University College of Medicine, 50-1 Yonsei-ro, Seodaemun-gu, 03722 Seoul, Republic of Korea; 2 Department of Internal Medicine, Yonsei University College of Medicine, Seoul, Republic of Korea

**Keywords:** Gastric cancer, Radiotherapy, Bleeding, Palliative treatment

## Abstract

**Background:**

Gastric bleeding negatively impacts the quality of life of patients with unresectable advanced gastric cancer and is frequently lethal. We investigated the efficacy of RT for palliation of gastric bleeding from gastric cancer and identified an optimal radiotherapy (RT) strategy.

**Methods:**

The study analyzed 57 patients submitted to palliative RT for gastric bleeding associated with gastric cancer between January 2009 and February 2019. Changes in hemoglobin (Hb) levels were analyzed based on measurements taken before and immediately, 1 month, and 2 months after RT. Re-bleeding after RT was identified as either Hb level dropping to < 7.0 g/dL or the administration of a blood transfusion after RT.

**Results:**

The median biologically effective dose (α/β = 10) was 37.5 Gy (range 23.6–58.5). The most common regimen was 25 Gy in five fractions. The mean Hb levels before, immediately after, 1 month, and 2 months after RT (6.6, 9.7, 10.3, and 9.7 g/dL, respectively) were significantly higher than that before RT (all p < 0.001). No significant differences in re-bleeding rates were observed according to total dose, fractional dose, and fraction number. Gastric tumor response evaluated by computed tomography within 2 months after RT showed partial responses were more frequent in patients achieving bleeding control (25.0% vs. 10.8%, p = 0.023) and overall survival was significantly improved for bleeding control within 3 months after RT (median, 15.4 vs. 10.0 weeks, p = 0.048).

**Conclusions:**

RT was an effective modality for gastric bleeding control in gastric cancer, which can be achieved with a short course scheme with five fractions.

**Supplementary Information:**

The online version contains supplementary material available at 10.1186/s13014-021-01884-5.

## Background

Gastric cancer accounted for 5.7% of all cancers worldwide in 2018, with Korea having the highest incidence; furthermore, gastric cancer is one of the most common causes of cancer-related death [1]. More than 10% of gastric cancer patients are associated with bleeding at the same time when they were first diagnosed, and more than half of them were major bleeding [2]. 5% of upper gastrointestinal bleedings are caused by tumor mass, with primary gastric cancer the most common cause of tumor bleeding [3, 4]. Bleeding caused by gastric cancer not only degrades patient quality but can also lead to life-threatening conditions due to hematological instability. This deterioration of patient condition also interferes with cancer treatment; thus, failure to control bleeding can lead to a poor prognosis [5].

Treatments for bleeding control include endoscopic therapy, transcatheter arterial embolization, and radiotherapy (RT). Endoscopic therapy showed hemostasis rates of 73–100%, making it excellent for bleeding control; however, the re-bleeding rates were approximately 40% [5–7]. In particular, predicted endoscopic hemostasis failure was higher for large bleeding lesions (> 2 cm) and non-exposed vessel bleeding [8]. Transcatheter arterial embolization also provides rapid control of massive bleeding. However, the re-bleeding rate after the first hemostasis is 41–66%, worse than that for endoscopic therapy [9, 10]. RT is also effective for tumor bleeding, with reported control rates of 50–89% [11–13]. RT is non-invasive compared to other treatments; hence, it can be used in patients with poor physical condition. However, the optimal palliative RT strategy has not been well-established due to a lack of conclusive data.

This study investigated the efficacy of RT for palliation of gastric cancer-associated gastric bleeding and suggested an optimal RT strategy.

## Methods

### Patients

The study analyzed 57 patients who received palliative RT for gastric cancer-associated gastric bleeding between January 2009 and February 2019 at Yonsei Cancer Center. In all patients, gastric bleeding was confirmed by esophagogastroduodenoscopy. Patients who neither had RT/gastric bleeding-related records or complete RT were excluded. We evaluated 57 patients who were eligible for our study.

This study was approved by the Severance Hospital institutional review board (No. 4-2020-1305), and the requirement for informed consent was waived because of the retrospective nature of this study. All methods were carried out in accordance with relevant guidelines and regulations.

### Radiotherapy

All patients underwent palliative RT using external-beam RT. The gross tumor volume was defined as the gross tumor lesion as seen on computed tomography (CT). Internal target volume was defined as considering the respiratory movement in the gross tumor volume, and four-dimensional CT with free breathing was used to account for the respiratory movement. Clinical target volume was defined as internal target volume plus 0.3 cm margins. Planning target volume was defined as clinical target volume plus 0.3 cm margins for set-up uncertainty. Dose was prescribed to planning target volume. The biologically effective dose (BED) was calculated to compare different RT fractionation regimens. BED was calculated according to the following equation: BED = nD[1 + D/(α/β)], where n = fraction number, D = dose per fraction, and α/β = 10 for gastric cancer.

### Response evaluation

Re-bleeding after palliative RT was defined as either a decrease in the hemoglobin (Hb) level to < 7.0 g/dL or blood transfusion after RT with or without an Hb level < 7.0 g/dL. The changes in Hb levels were analyzed at various time points: the baseline Hb within 1 month before initiating RT, Hb at RT completion, at 1 month, and 2 months after RT completion.

We also assessed whether our patients no longer had clinical symptoms (melena, hematemesis) after RT. These clinical symptoms were confirmed through the medical record, and it was mainly checked whether the symptoms were relieved one month after the end of RT.

In patients who underwent endoscopy after RT, we checked whether the bleeding improved on the endoscopic findings.

To evaluate the response of gastric tumors by RT, CT images were performed within 1 to 2 months after the end of RT. Tumor response was determined according to Response Evaluation Criteria in Solid Tumors (RECIST), version 1.1 [14]. Treatment-related toxicities were graded according to the Criteria for Adverse Events, version 5.0 [15].

### Statistical analysis

Cumulative re-bleeding rate (CRR) was defined as the time from RT completion to Hb level dropping below 7.0 g/dL or receiving a blood transfusion after RT with or without Hb level < 7.0 g/dL. Overall survival (OS) was defined as the time from RT completion to death or last patient contact. CRR and OS were analyzed using the Kaplan–Meier method and log-rank test. Univariate and multivariate analyses for CRR were performed with the Cox proportional hazards model.

Repeated-measures analysis of variance (ANOVA) was performed to assess the changes in Hb level at each time point after RT (the baseline Hb within 1 month before initiating RT, Hb at RT completion, at 1 month, and 2 months after RT completion).

The Pearson χ2 test was used to identify the relationship between the gastric tumor response and bleeding control after RT.

P-values lower than 0.05 were considered statistically significant. Statistical analysis was performed using IBM SPSS Statistics for Windows, version 23.0 (IBM Corp., Armonk, NY, USA).

## Results

### Patient and treatment characteristics

The baseline characteristics of all patients are listed in Table 1. The median age was 61 years (range, 33–87 years). Fifty-two patients (91.2%) and 5 patients (8.8%) were diagnosed with adenocarcinoma and signet ring cell carcinoma, respectively. Fifty patients (87.7%) had distant metastasis at the time of RT and 7 (12.3%) patients had locally advanced disease.

**Table 1:** Patient and tumor characteristics

Characteristic	N (%)
Age (median years, range)	61 (33–87)
Sex	
Male	35 (61.4)
Female	22 (38.6)
Performance status (Eastern Cooperative Oncology Group)	
1	26 (45.6)
2	21 (36.8)
3	8 (14.0)
4	2 (3.5)
Histopathology	
Adenocarcinoma	52 (91.2)
Signet ring cell carcinoma	5 (8.8)
Stage (American Joint Committee on Cancer 8th revision)	
IIA	1 (1.8)
IIB	2 (3.5)
IIIA	2 (3.5)
IIIB	2 (3.5)
IV	50 (87.7)
Chemotherapy	
Before radiotherapy	43 (75.4)
During radiotherapy	10 (17.5)
After radiotherapy	27 (47.4)
Endoscopic hemostasis therapy before radiotherapy	
No	11 (19.3)
Yes	46 (80.7)
Baseline hemoglobin (median g/dL, range)	6.6 (3.9–9.9)

**Fig. 1:**
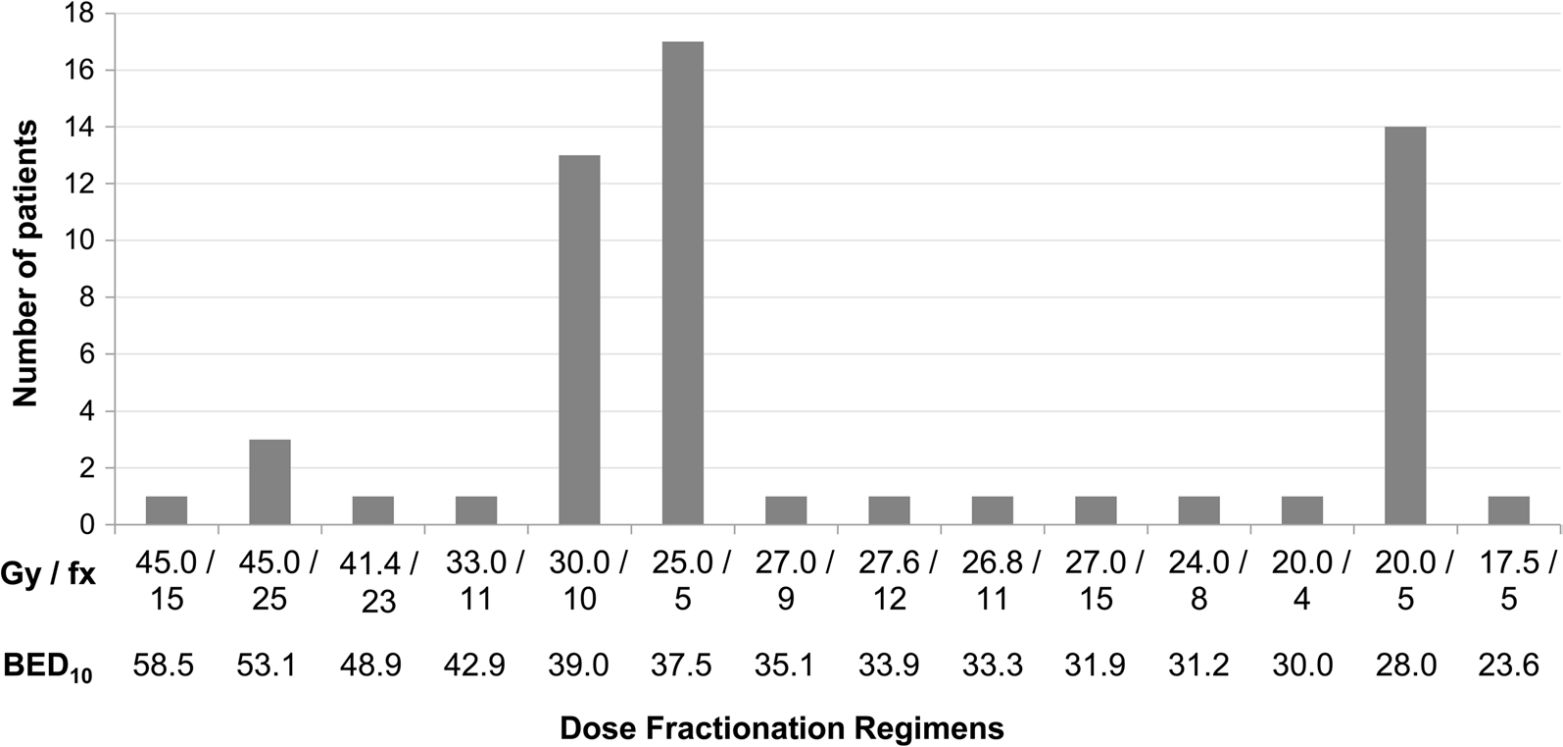
Histogram depicting the distribution of radiation doses for the treatment of gastric bleeding

All but one patient (98.2%) received three-dimensional conformal RT (3D-CRT). The remaining one patient received intensity modulated RT (IMRT). The median total applied dose was BED_10_ 37.5 Gy (range 23.6–58.5 Gy). All patients have completed scheduled RT. The median dose per fraction was 4.0 Gy (range 1.8–5.0 Gy) and a median of 5 fractions (range, 4–25 fractions). The most common dose fractionation regimen was 25 Gy in five fractions (29.8%), followed by 20 Gy in 5 fractions (24.6%) and 30 Gy in 10 fractions (22.8%) (Fig. 1).

Forty-three patients (75.4%) received standard dose of chemotherapy before RT, 10 patients (17.5%) during RT, and 27 patients (47.4%) after RT. Ten patients received irinotecan plus folinic acid/fluorouracil, 11 patients received oxaliplatin plus folinic acid/fluorouracil, 11 patients received capecitabine plus oxaliplatin, and 11 patients received S-1 plus cisplatin. There were 46 patients (80.7%) who underwent endoscopic hemostatic treatment before RT.

### Changes in symptoms and transfusion

Forty-three patients (75.4%) showed subjective symptom improvements one month after the end of RT; melena in 27 patients, hematemesis in 10 patients, and both in 6 patients. The remaining 14 patients (24.6%) continued to have subjective symptoms after RT. Among these 14 patients, eight continued to have melena, two continued bleeding as observed through the Levin Tube, and four had recurrent hematemesis.

All but one patient received blood transfusions before RT, and all of them received transfusions within 1 week prior to initiation of RT. There were 50 patients (87.7%) who received blood transfusions during RT. After RT, 21 patients (36.8%) received no further transfusions. The median interval from the end of RT to receiving transfusion was 2.9 weeks (range, 0.3–38.9 weeks) in 36 patients who received transfusion even after RT.

### Endoscopic assessment

Twenty-one patients (36.8%) underwent endoscopy within 2 months after RT completion. Of these, 14 (66.7%) were confirmed to stop bleeding completely. Five (23.8%) had minor bleeding like oozing, and the other 2 (9.5%) had no response. No patients were found to have new bleeding in another point other than the treated.

### Changes in Hb level

**Fig. 2:**
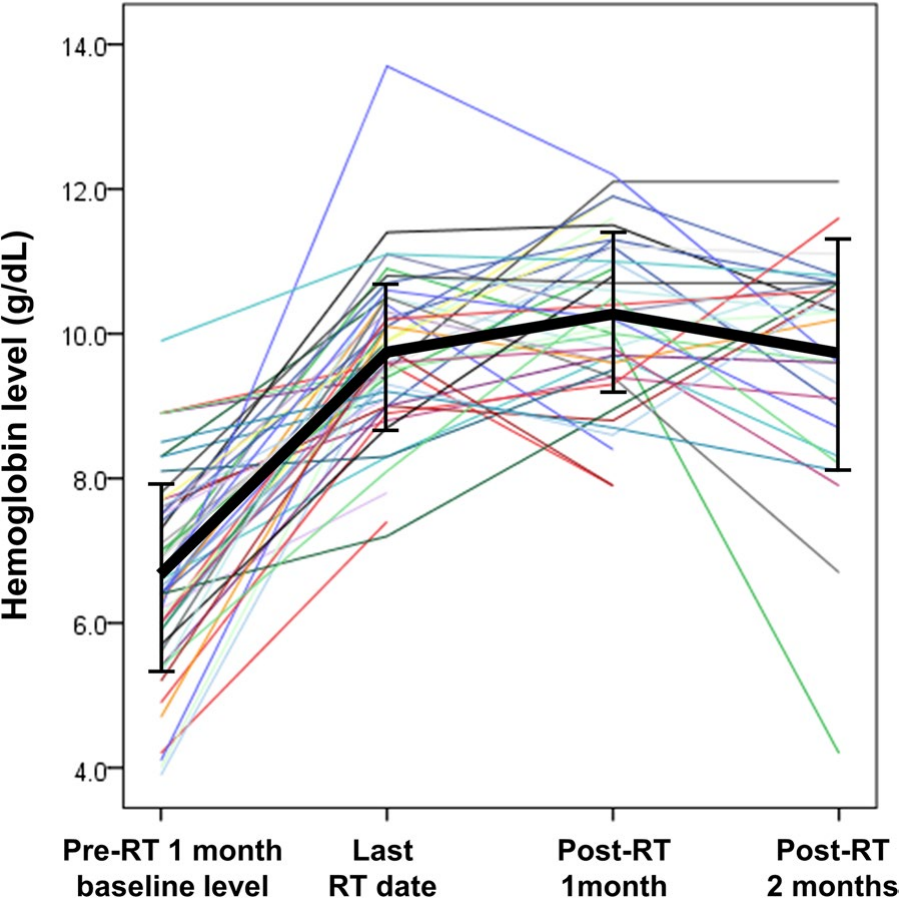
Changes in hemoglobin levels in individual patients (thin colored line) and mean hemoglobin levels of patients (bold black line)

The mean of the baseline Hb level within 1 month before RT was 6.6 ± 1.3 g/dL, while the mean levels immediately, 1 month, and 2 months after RT were 9.7 ± 1.0 g/dL, 10.3 ± 1.1 g/dL, and 9.7 ± 1.6 g/dL, respectively. The changes in Hb levels and mean patient Hb levels are shown in Fig. 2. Comparison of the mean of the baseline Hb level within 1 month before RT to the mean values at each time point (immediately, 1 month, and 2 months after RT) showed significant increases for all (p < 0.001, respectively).

Thirty-five patients (61.4%) had the baseline Hb level within 1 month before RT below 7.0 g/dL. All patients had Hb levels above 7.0 g/dL immediately and 1 month after RT. At 2 months after RT completion, only two patients had Hb levels below 7.0 g/dL.

Of the 35 patients with the baseline Hb level within 1 month before RT below 7.0 g/dL, 14 (38.9%) had levels below 7.0 g/dL at least once during the follow-up period after RT completion. In contrast, of the 22 patients with the baseline Hb level within 1 month before RT above 7.0 g/dL, 5 (23.8%) had Hb levels below 7.0 g/dL at least once during the follow-up period after RT.

### Re-bleeding free rate and toxicity

**Fig. 3:**
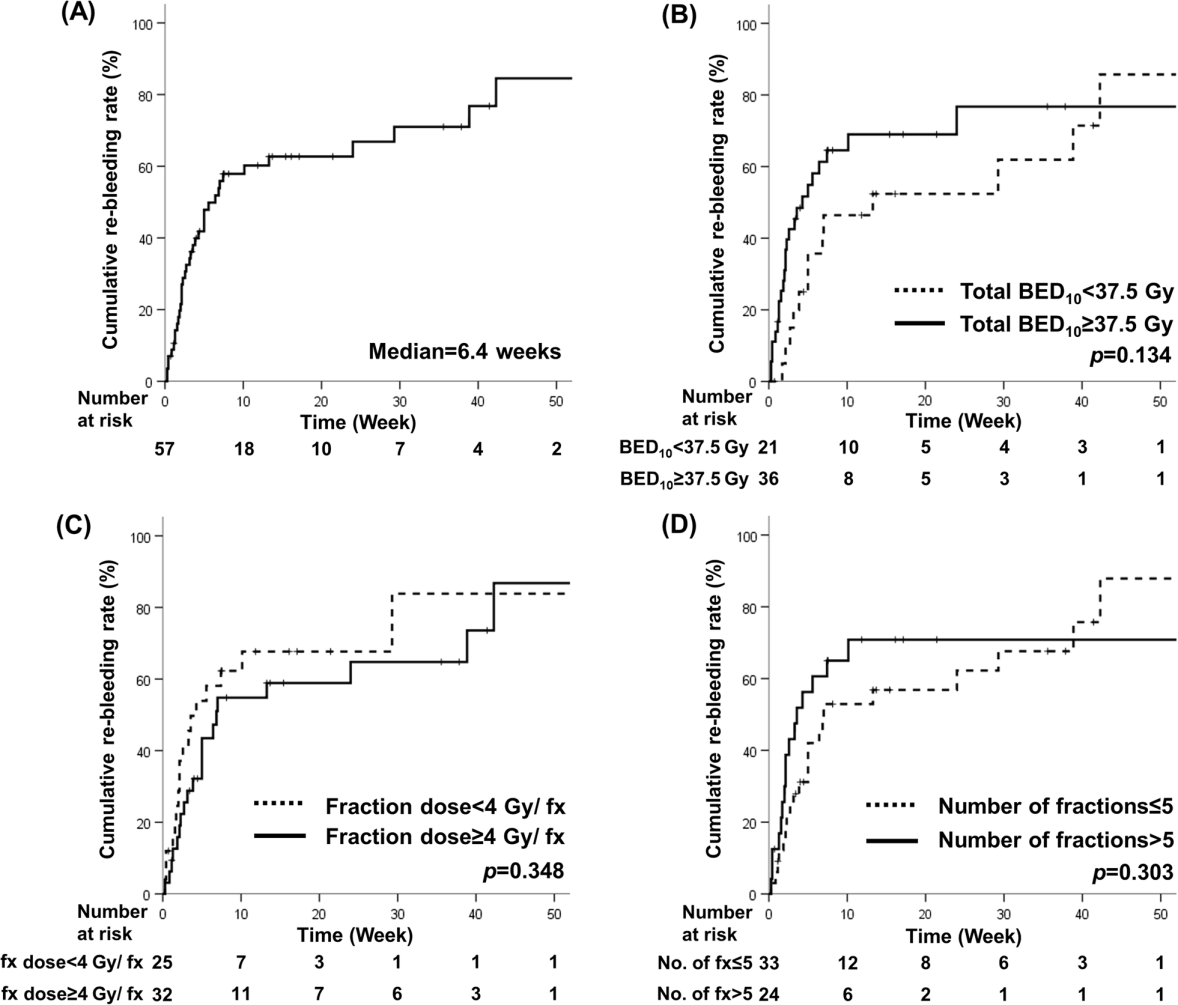
Cumulative re-bleeding rates for **a** all patients and by **b** total dose, **c** dose per fraction, and **d** number of fractions

The median bleeding-free duration after RT was 6.4 weeks (range 0.3–237.7). CRR at 3 months after RT was 60.2% (Fig. 3a). CRR was analyzed according to median value of several factors in RT but showed no significant differences for total BED_10_ 37.5 Gy (3 months, CRR 46.4% vs. 69.0%, p = 0.134), fraction dose 4 Gy (3 months, CRR 67.7% vs. 54.8%, p = 0.348), or fraction number 5 (3 months, CRR 52.9% vs. 70.8%, p = 0.303) (Fig. 3). In univariate analysis, younger age (hazard ratio 0.97, 95% confidence interval 0.95–0.99, p = 0.010) and signet ring cell carcinoma histology (hazard ratio 4.46, 95% confidence interval 1.62–12.26, p = 0.004) were identified poor predictive factors for CRR. However, multivariate analysis showed no independent risk factors associated with CRR (Additional file 1).

The gastric tumor response was evaluated in 37 patients by CT scan within 2 months after RT. Four of the 37 (10.8%) patients had gastric tumor progression despite RT, while 24 (64.9%) had stable disease and nine (24.3%) had a partial response. Partial responses were observed more frequently in patients with controlled bleeding (25.0% vs. 10.8%, p = 0.023) (Table 2).

**Table 2:** The relationship between bleeding control and tumor response

Tumor response	Number of controlled patients (%)	Number of non-controlled patients (%)
Partial response	5 (25.0)	4 (10.8)
Stable disease	4 (20.0)	20 (54.1)
Progressive disease	0 (0.0)	4 (10.8)
Non-evaluated	11 (55.0)	9 (24.3)
Total	20	37

Among reported treatment-related toxicities, 28 (49.1%) and 2 (3.5%) patients reported grade 1 and 2 toxicity, respectively. No grade 3 or higher toxicities were reported.

### Survival

**Fig. 4:**
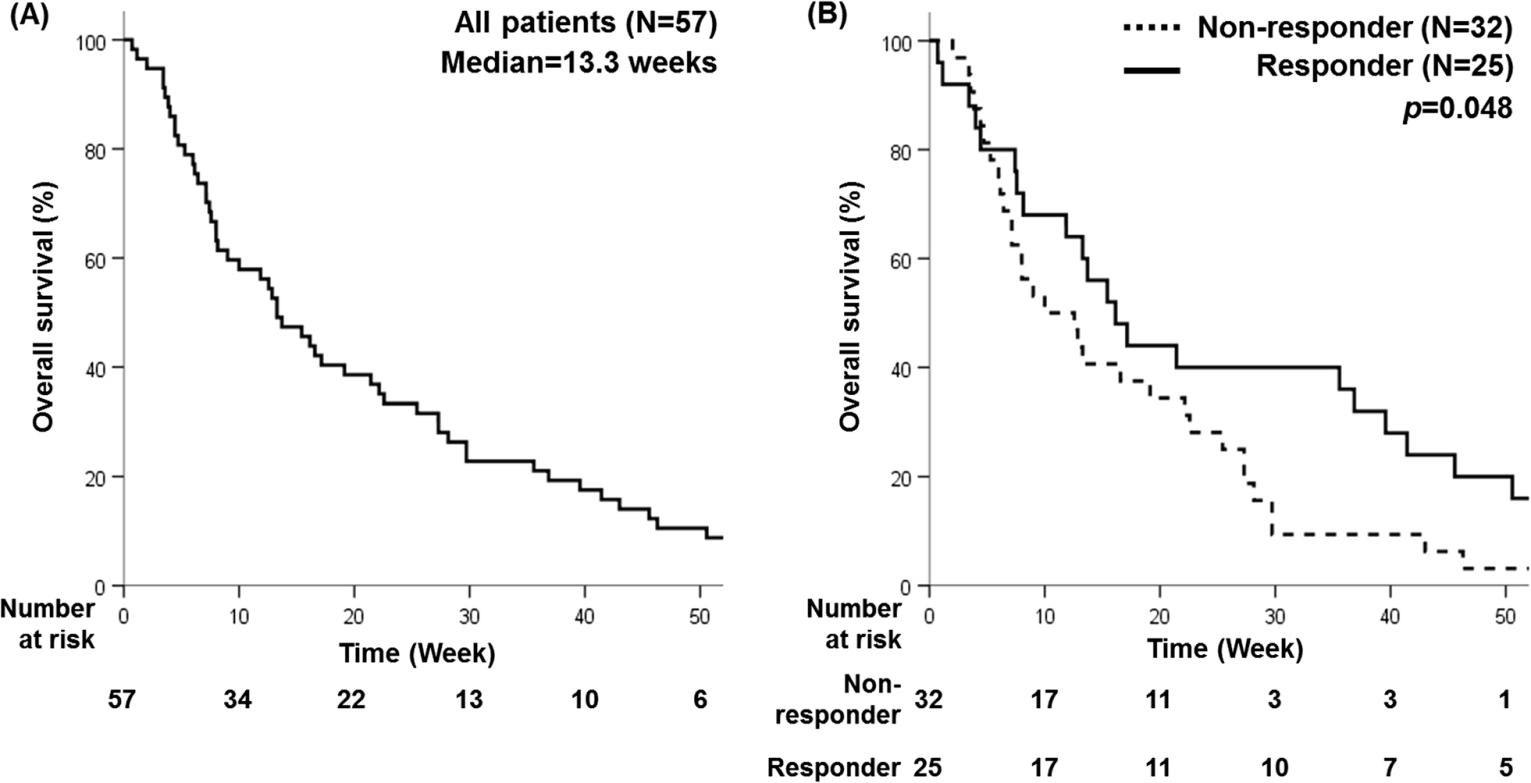
Overall survival for (**a**) all patients and (**b**) by response to radiotherapy

Over a median follow-up duration of 13.3 weeks (range 0.7–237.7), 53 patients died (Fig. 4a). Patients whose bleeding was controlled within 3 months after RT completion showed a significantly improved OS (p = 0.048) (Fig. 4b), at 15.4 weeks in 25 patients without re-bleeding vs. 10.0 weeks in 32 patients with re-bleeding.

## Discussion

The results of this study showed that palliative RT is an effective modality for gastric bleeding control in gastric cancer. Although the Hb level before RT was low (mean, 6.6 g/dL), the level after RT increased to more than 9.7 g/dL. A bleeding-free duration of 6.4 weeks was achieved with palliative RT without no grade 3 or 4 toxicities. Tumor response was significantly better in patients without re-bleeding and OS was significantly higher in patients whose bleeding was controlled within 3 months after RT completion.

Previous studies showed that RT is an effective modality for gastric bleeding control in gastric cancer. Since there is no established dose regimen for palliative RT, these studies administered various regimens, with fraction doses ranging from 1.8 to 8 Gy and total doses from 6 to 60 Gy, corresponding to BED_10_ of 7.2–50.8 Gy [11–13, 16–19]. Few studies have compared differences in treatment outcomes by fraction dose or numbers. Sapienza et al. showed that the fraction number (> 5 vs. ≤5) was not significantly associated with bleeding control [20]. In line with previous reports, our study showed no differences in re-bleeding rates according to fraction dose and number.

Several studies have assessed the correlation between total dose and bleeding controls. One systematic review suggested similar bleeding control effects between low and high BED regimens [21]. In contrast, several studies suggested that higher BED regimens are more effective [12, 16, 22], with BED_10_ over 36–41 Gy showing significantly better local control. In our study, patients were administered various regimens, with total dose BED_10_ of 23.6–58.5 Gy and fraction doses of 1.8–5.0 Gy. While short-course schemes were preferred for better compliance, protracted courses were selected for patients with large-volume tumors due to concerns regarding toxicity. However, efficacy appeared similar regardless of the fractionation scheme, without toxicity. Therefore, short fractionation regimens with high fractional doses may be reasonable in patients with poor performance status or limited life expectancy and those requiring urgent symptom control. However, for large tumor volumes, it may be better to flexibly administer 30 Gy in 10-fraction regimens.

Due to the difficulty in defining objective parameters, no standard has yet been established for evaluation of the effect of bleeding control after RT. The most obvious method is to check for hemostasis directly through endoscopic examinations, but quite a number of patients with gastric bleeding have failed to perform post-RT endoscopic examinations due to reasons such as poor condition. In our study, 63.2% of patients failed to perform endoscopy after RT. Like previous reports, we also used clinical symptoms (melena and hematemesis) to evaluate the hemostatic effect of RT [12, 16, 23] and observed clinical symptom relief in 75.4% of cases. Other studies evaluated the response to RT by reviewing the pre- and post-RT Hb levels as well as the reduction in numbers of transfused units or volume of transfused blood after RT [24, 25]. Hb is an objective measure of bleeding; we also showed a significant increase in mean Hb level after RT. However, Hb level does not directly reflect acute gastric bleeding and the individual baseline Hb levels varied widely according to underlying comorbidities and chemotherapy. Therefore, single Hb measurements may be undesirable as a standard evaluation tool for assessing treatment results. In addition, the number of blood transfusions does not directly reflect bleeding control because of its subjectivity.

Our study proposed two criteria for evaluating re-bleeding. First, we observed whether the Hb level dropped below 7.0 g/dL after RT. This cutoff is considered a threshold value in restrictive transfusion strategies recommended for upper gastrointestinal bleeding [26]. In our study, no patients had Hb levels below 7.0 g/dL until one month after RT and only two patients received blood transfusions because their Hb levels dropped below 7.0 g/dL at 2 months after RT. Second, we observed if the bleeding was confirmed or blood transfusion was administrated even when Hb levels did not fall below 7.0 g/dL. Our criteria aimed to include both Hb level and transfusion in bleeding control evaluation and we defined re-bleeding as meeting one of the two criteria, different from the definitions used in other studies.

In this study, the Hb level at the immediately end of RT was significantly higher than the lowest Hb level within 1 month before RT (Fig. 2). This might be related to that most of our patients received transfusions within 1 week prior to initiation of RT and during RT. However, it is noteworthy that the number of patients receiving transfusions after RT has significantly decreased. In addition, the Hb level continued to rise until a month after the end of RT, which suggests bleeding control through RT. Therefore, RT seems to keep the Hb elevation effect of transfusions as long as possible and to further eliminate additional transfusions.

We also analyzed the association between re-bleeding and OS. The reported duration of response varied greatly, with a median ranging from 1.5 to 11.4 months [21]. Lee et al. also proposed the use of mean Hb level and reduction value in transfusion units within 3 months after RT [23]. According to our data, patients whose bleeding was controlled within 3 months after RT completion showed significantly improved OS.

One patient had a baseline Hb concentration of 12–15 g/dL, which dropped to 9.9 g/dL. Bleeding was confirmed by endoscopy. After RT, the Hb level increased to 11 g/dL. Although this patient did not have significant anemia initially, they received RT due to the significant drop in Hb level from baseline. As such, RT can be administered flexibly at physicians’ discretion even when Hb level criteria are not met. Another patient had a drop in Hb level to 4.2 g/dL at 2 months post-RT, which was the baseline Hb level observed in our study. This patient had a diffuse gastric cancer lesion on the entire stomach wall and, thus, received RT to the whole stomach, with a dose of 30 Gy in 10 fractions. The initial Hb level was 6.9 g/dL, which increased to 10.0 g/dL at 1 month post-RT. However, at 2 months post-RT, the level decreased to 4.2 g/dL, with melena re-occurrence. Thus, reirradiation of 20 Gy in 10 fractions was administered, with no toxicity above grade 2. Bleeding control was also successful, with melena disappearing and Hb maintained at over 9.0 g/dL until 1 month post-RT. Thus, reirradiation is also an acceptable option when the first palliative attempt fails.

Due to its retrospective nature, this study has some limitations. The RT schemes were varied according to physician’s preference although some tendency existed using a short-course scheme in case large tumor size or poor condition of the patients. So there might be selection bias. Therefore, in the future, it seems necessary to plan a prospective study to reduce treatment-related errors by specifying a prescription scheme of RT. Moreover, post-RT additional treatment involving chemotherapy, which might have affected prognosis, couldn’t be fully analyzed. Further study with larger population is needed to clarify this issue. Finally, quality survey such as European Organization for Research Treatment of Cancer (EORTC) QLQ-C30 [27], which could provide more objective evaluation, was not implemented in this study due to retrospective nature. To compensate this, we reviewed each case in detail and checked the symptoms at a consistent time point after the end of RT.

## Conclusions

In conclusion, RT was an effective and well-tolerated modality for gastric bleeding control in gastric cancer regardless of the fractionation regimen, resulting in significantly increased Hb levels. In practical terms, the use of a short-course scheme with five fractions is a suitable option for patients with poor performance status or limited life expectancy, as well as those requiring urgent symptom control.

## Supplementary Information

**Additional file 1**.** Supplementary Table 1**. Univariate and multivariate analysis for cumulative re-bleeding rate.

## Data Availability

The datasets used and/or analyzed during the current study are available from the corresponding author on reasonable request.

## Abbreviations

RT: Radiotherapy
CT: Computed tomography
BED: Biologically effective dose
Hb: Hemoglobin
RECIST: Response Evaluation Criteria in Solid Tumors
CRR: Cumulative re-bleeding rate
OS: overall survival
ANOVA: Analysis of variance
3D-CRT: Three-dimensional conformal radiotherapy
IMRT: Intensity modulated radiotherapy
EORTC: European Organization for Research Treatment of Cancer
