# Supplementary material for: Efficacy of radiotherapy for gastric bleeding associated with advanced gastric cancer
Source: Radiat Oncol. 2021 Aug 23;16:161. doi: 10.1186/s13014-021-01884-5 (PMC8383356; doi:10.1186/s13014-021-01884-5)
Supplement: Supplementary file 1 — Additional file 1. Supplementary Table 1. Univariate and multivariate analysis for cumulative re-bleeding rate. [file 13014_2021_1884_MOESM1_ESM.docx]

Supplementary Table 1. Univariate and multivariate analysis for cumulative re-bleeding rate

|  | Univariate analysis | | Multivariate analysis | |
| --- | --- | --- | --- | --- |
|  | HR (95% CI) | p-value | HR (95% CI) | p-value |
| Age^*^ | 0.97 (0.95-0.99) | 0.010 | 0.98 (0.94-1.01) | 0.162 |
| Sex (Male vs. Female) | 1.38 (0.71-2.69) | 0.345 | 1.91 (0.80-4.57) | 0.148 |
| Performance status (ECOG) |  |  |  |  |
| 1 | 1.00 |  | 1.00 |  |
| 2 | 1.65 (0.81-3.39) | 0.169 | 2.48 (0.90-6.82) | 0.078 |
| 3 | 1.43 (0.52-3.92) | 0.491 | 2.31 (0.70-7.68) | 0.172 |
| 4 | 0.99 (0.13-7.49) | 0.990 | 0.86 (0.09-8.32) | 0.896 |
| Histopathology  (Adenocarcinoma vs. Signet ring cell carcinoma) | 4.46 (1.62-12.26) | 0.004 | 3.16 (0.70-14.35) | 0.136 |
| T stage (T2-3 vs. T4) | 1.21 (0.62-2.35) | 0.572 | 1.36 (0.62-2.97) | 0.438 |
| Chemotherapy before RT (No vs. Yes) | 1.96 (0.83-4.61) | 0.126 | 1.59 (0.50-5.01) | 0.432 |
| Chemotherapy during RT (No vs. Yes) | 0.84 (0.35-2.02) | 0.691 | 1.73 (0.56-5.36) | 0.344 |
| Chemotherapy after RT (No vs. Yes) | 1.05 (0.55-2.02) | 0.882 | 0.85 (0.36-2.03) | 0.721 |
| Endoscopic hemostatic treatment before RT (No vs. Yes) | 0.80 (0.33-1.93) | 0.612 | 0.80 (0.24-2.63) | 0.713 |
| Total BED_10_^*^ | 1.03 (0.98-1.07) | 0.240 | 1.07 (0.97-1.19) | 0.188 |
| Fraction dose^*^ | 1.00 (1.00-1.00) | 0.876 | 1.00 (0.99-1.01) | 0.293 |
| Fraction number^*^ | 1.01 (0.95-1.07) | 0.822 | 0.87 (0.67-1.12) | 0.273 |
| PTV size^*^ | 1.00 (1.00-1.00) | 0.540 | 1.00 (1.00-1.00) | 0.928 |

* Age, total BED_10_, fraction dose, fraction number, and PTV volume was treated as a continuous variable

The foreparts of the parentheses were set as the reference groups in the multivariable analysis

***Abbreviations:*** HR, hazard ratio; CI, confidence interval; ECOG, Eastern Cooperative Oncology Group; RT, radiotherapy; BED_10_, biologically effective dose (α/β=10); PTV, planning target volume
